# Management of provoked seizure

**DOI:** 10.4103/0972-2327.78041

**Published:** 2011

**Authors:** Usha Kant Misra, Jayantee Kalita

**Affiliations:** Department of Neurology, Sanjay Gandhi Post Graduate Institute of Medical Sciences, Lucknow, India

**Keywords:** Alcohol, cerebral venous sinus thrombosis, liver failure, porphyria, provoked seizure, renal failure, stroke, traumatic brain injury

## Abstract

A provoked seizure may be due to structural damage (resulting from traumatic brain injury, brain tumor, stroke, tuberculosis, or neurocysticercosis) or due to metabolic abnormalities (such as alcohol withdrawal and renal or hepatic failure). This article is a part of the Guidelines for Epilepsy in India. This article reviews the problem of provoked seizure and its management and also provides recommendations based on currently available information. Seizure provoked by metabolic disturbances requires correction of the triggering factors. Benzodiazepines are recommended for treatment of seizure due to alcohol withdrawal; gabapentin for seizure seen in porphyria; and antiepileptic drugs (AED), that are not inducer of hepatic enzymes, in the seizures seen in hepatic dysfunction. In severe traumatic brain injury, with or without seizure, phenytoin (PHT) may be given for 7 days. In ischemic or hemorrhagic stroke one may individualize the AED therapy. In cerebral venous sinus thrombosis (CVST), AED may be prescribed if there is seizure or computed tomographic (CT) abnormalities or focal weakness; the treatment, in these cases, has to be continued for 1 year. Prophylactic AED is not recommended in cases of brain tumor and neurosurgical procedures and if patient is on an AED it can be stopped after 1 week.

## Introduction

Provoked seizures are those seizures occurring within 7 days of acute brain insult. A large number of clinical conditions can result in seizures; they can be broadly categorized into two groups:

- Structural abnormalities caused by head injury, neurosurgical intervention, stroke, central nervous system (CNS) infections (e.g., neurocysticercosis, tuberculoma, and encephalitis)
- Metabolic/toxic disturbances, e.g., as seen in alcohol withdrawal and liver and kidney failure.

The high frequency of the triggering events that lead to provoked seizures and the paucity of controlled trials make a scientific evidence-based approach difficult, but some common subjects are summarized in this review.

## Traumatic Brain Injury

### Evidence statement

*Severe TBI*: Head injury results in seizure in 2–12% cases. Seizure is more frequently seen in severe (12%) or penetrating head injury (>50%).[1–3] Seizure appearing 1 h after a severe head injury (i.e., head injury with impairment of consciousness for 12–24 h, amnesia, depressed fracture of skull, and contusion or hematoma) increases the risk of recurrence of seizure. Analysis of two class I studies reveal that phenytoin (PHT) significantly reduces the rate of early posttraumatic seizures in severe TBI within 7 days.[4,5] Pooled evidence from class I and II studies in severe TBI reveal that there is no benefit of PHT, sodium valproate (SVA), and carbamazepine (CBZ) in preventing late seizures after 7 days. Patients with severe TBI should receive intravenous PHT to prevent posttraumatic seizure within 7 days and PHT should not be continued after 7 days.[6]

*Mild TBI*: In mild TBI (i.e., head injury with brief loss of consciousness of less than 30 min, headache, vomiting, and amnesia), patients have only a slightly increased risk of developing posttraumatic seizures, including early posttraumatic seizures (<1 week). Prophylactic anticonvulsants are not indicated. A systematic review of 2036 patients showed that prophylactic anticonvulsant treatment did not reduce mortality, neurological disability, or late seizures.[7] If recurrent seizures occur, treatment is probably indicated and an alternate explanation such as delayed hematoma, Wernicke-Korsakoff syndrome, alcohol withdrawal, or electrolyte disturbance should be considered.

*Minor head injury in children*: In children with minor closed head injury without loss of consciousness and with normal neurological examination, the chances of finding an abnormality on CT is low and, therefore, CT scan is not routinely recommended. These children should be observed for 12–48 h by an adult caregiver who can monitor the child and seek help when necessary. In children with minor head injury, with loss of consciousness of less than 1 min and a normal physical and neurological examination, CT scan may be done. Those with a normal CT scan may be discharged or observed as mentioned above. Prophylactic AED is not recommended. Children with asymptomatic intracranial hemorrhage (subdural hemorrhage or intracerebral hemorrhage) should be observed rather than operated upon.[8] Electroencephalography (EEG) has a limited role as a predictor of risk of posttraumatic seizures. In one study, of those patients with a normal EEG at 3 months, 20% developed seizure[9] and of those with normal EEG at 1 month, 8.3% developed partial seizures and 27.3% generalized seizures.[10]

### Recommendation

Patients with severe TBI should receive intravenous PHT to prevent posttraumatic seizure within 7 days; PHT should not be continued after 7 days. In mild TBI, antiepileptic prophylaxis is not recommended (class I, level A).

## Neurosurgery

### Evidence statement

There are three major indications for AEDs in neurosurgical practice: (a) head injury (already discussed), (b) brain tumor (before or after a brain surgery), and (c) patients undergoing radiotherapy or chemotherapy.

Seizures are reported in more than one-third of patients with primary brain tumors. The incidence of seizure varies depending on the location of the tumor and the histologic subtype. In those patients who have had a seizure, anticonvulsant therapy is obviously recommended. Patients who have not had a seizure have a 20–45% risk of developing one. Patients with brain tumors often receive prophylactic AEDs to prevent seizure. A meta-analysis of 12 studies – either randomized controlled studies (RCTs) or cohort studies that investigated the use of prophylactic anticonvulsants (PHT, phenobarbitone, or SVA) in patients with primary and metastatic brain tumors – demonstrated a lack of efficacy of these drugs in preventing the first seizure or recurrence of the seizures.[11]

Six prospective, controlled trials reviewed by Temkin and his colleagues[12] showed that, when patients with brain tumors undergo surgery, prophylactic treatment reduces the risk for early seizures by 40–50%, as compared with placebo or no treatment, but it does not reduce the incidence of late seizures. Similar findings were also obtained in patients undergoing craniotomy for a variety of other conditions, including vascular malformations and abscesses. A meta-analysis of RCTs done to evaluate the role of AED prophylaxis after supratentorial surgery favored the use of PHT to prevent early seizures, the relative risk being 0.42. However, there was no evidence that long-term treatment with PHT or CBZ could protect against late seizures.[12]

On the basis of the above data, the following recommendations have been made:[11]

- In patients with newly diagnosed brain tumors anticonvulsant medications are not effective in preventing first seizures and should therefore be avoided.
- In patients with brain tumors who have not had a seizure, tapering and discontinuing anticonvulsants after the first postoperative week is appropriate. However, if surgery is followed by radiation therapy, it has been suggested that continued prophylactic treatment beyond the first week may be useful.

Despite the confounding effect of the underlying cerebral pathology and surgery, radiotherapy of the brain may increase the risk for seizures, either on a short-term basis through direct brain damage caused by the procedure or in the long term as a result of radiotherapy-induced cerebral vasculopathy.[13] Many centers recommend maintenance of AED prophylaxis as long as radiotherapy is continued.

During radiotherapy, aromatic AEDs such as phenobarbitone, PHT, and CBZ result in a high frequency of Steven-Johnson syndrome or toxic epidermonecrolysis; therefore SVA, gabapentin, and levetiracetam are recommended (class IV).

Enzyme-inducing AEDs such as PHT, phenobarbitone, and CBZ increase the clearance and reduce the clinical efficacy of anticancer agents such as nitrogen mustards, vinca alkaloids, anthracyclines, taxanes, epipodophyllotoxins, and alkylating agents, which are also metabolized by the cytochrome isoenzymes. Several investigators have reported decrease in the concentrations of taxanes, vinca alkaloids, methotrexate, teniposide, or camptothecin analogues in patients receiving enzyme-inducing AEDs, thus indicating the need to increase their dose in such situations.[14,15] A deleterious effect of anticonvulsants on the efficacy of anticancer agents has also been reported. Significantly worse event-free survival, hematologic relapse rate, and CNS relapse rate were reported in children with acute lymphoblastic leukemia who received teniposide, methotrexate, and enzyme-inducing AEDs.[16] In contrast, enzyme-inhibiting AEDs such as SVA may impair the metabolism of chemotherapeutic agents, thereby increasing toxicity. SVA has been reported to increase the incidence of thrombocytopenia and neutropenia in patients with high-grade glioma who were treated with a combination of nitrosourea, cisplatin, and etoposide.[17] Chemotherapeutic agents may also affect the pharmacokinetics of AEDs. Cisplatin in association with carmustine and vinblastine with methotrexate have been reported to reduce the PHT levels in patients with primary brain tumors. Likewise, treatment with doxorubicin and cisplatin may reduce CBZ and SVA levels.[18] Methotrexate has also been reported to reduce serum SVA concentrations by 25%. On the other hand, 5-fluorouracil plus leucovorin[19] and high-dose tamoxifen increased PHT concentrations.[20]

### Recommendation

AEDs should not be used for preventing the first seizure in neurosurgical patients; if the patient is on AED, it should be stopped after 1 week (class I, level A). During radiotherapy, aromatic AEDs such as phenobarbitone, PHT, and CBZ result in high frequency of Steven-Johnson syndrome or toxic epidermonecrolysis; therefore SVA, gabapentine, and levetiracetam are recommended in this situation (class IV).

## Stroke

### Evidence statement

Stroke results in a 23- to 35-fold increase in seizure incidence[21–23] and the risk of epilepsy increases 17-fold.[24] Hemorrhagic stroke, subarachnoid hemorrhage, and cerebral venous sinus thrombosis (CVST) are associated with a higher risk of early seizures than is ischemic stroke.[25,26] The frequency of late seizure or epilepsy following cerebral hemorrhage is 2.7% and that after infarction is 3.8%.[27] Remote epilepsy has been reported in 5–26.6% patients with CVST.[28,29] There has been no RCT on the use of AEDs for control of post-stroke seizure in ischemic or hemorrhagic stroke nor is there evidence to support their use for primary prevention of post-stroke seizure or in CVST.[30,31] The risk of seizure after stroke is probably the same as in the general population after a first unprovoked seizure,[32,33] and withholding AEDs till a second seizure occurs may not be harmful.[34] Therefore, the decision about starting AED should be individualized on the basis of the characteristics of the first seizure and patient preference. First-line AEDs can interfere with recovery from stroke, interact with other drugs (such as other AEDs, anticoagulants, antiplatelet drugs, and statins), and can adversely affect bone health; therefore, the selection of the appropriate AED should be based on these considerations as well. There is no consensus about the ideal AED. Only gabapentin has been prospectively evaluated in post-stroke seizures; this showed that 81% of patients were seizure free at a median follow-up of 30 months.[35,36] Level A evidence is present only for lamotrigine and gabapentin as first-line antiepileptic drugs for treatment of partial-onset seizure in the elderly.[37] Low doses (100 mg) of lamotrigine and slow-release formulations of carbamazepine (400 mg) have also been found to be equally effective and can be cheaper alternatives.[38] A recent trial comparing lamotrigine and CBZ as monotherapy in elderly post-stroke patients suggested that lamotrigine is better tolerated, with a trend towards higher efficacy.[39]

AED prophylaxis is also controversial in CVST and no RCT has been carried out on this subject. However, the presence of a focal neurological deficit or a focal parenchymal lesion on CT/MRI and the presence of seizure is indication for treatment with an intravenous (IV) bolus of phenytoin followed by oral AED for at least 1 year.[40] In a study on 624 CVST patients with supratentorial lesions and those with presenting seizures predicted the occurrence of early seizure. The risk of early seizures in patients with supratentorial lesions and seizure at presentation was significantly lower when AED prophylaxis was used. For the prevention of remote seizures, AEDs are recommended for patients with seizures in the acute phase and for those who experience a seizure after the acute phase. These drugs can also be considered for patients without seizures who have supratentorial hemorrhagic lesions or motor deficits.[41]

### Recommendation

In the absence of class I studies, prophylactic AED is not recommended in hemorrhagic and ischemic stroke. After the first seizure, one may individualize the treatment or wait for the second seizure and use slow-release carbamazepine. In CVST, the presence of seizure, a focal deficit, or a focal cortical lesion on CT/MRI is indication for treatment with an IV phenytoin bolus followed by oral AED for 1 year (class III, level B).

## Alcohol-related Seizures

### Evidence statement

Alcohol withdrawal seizures are common and are diagnosed on the basis of the patient’s drinking history as reported by friends or relatives. The majority (90%) of alcohol withdrawal seizures occur within 48 h of withdrawal. These usually occur singly or in brief clusters. Status epilepticus occurs in less than 10% of cases. The great majority of alcohol withdrawal seizures are generalized; a focal onset suggests an intracranial structural lesion. If seizures occur after 48 h, other possibilities such as head injury should be considered. The diagnostic yield of CT scan after the first alcohol-related seizure is high because of the presence of structural lesions; however, for recurrent alcohol-related seizures, CT is not indicated. Change in type and frequency of seizure after 48 h of drinking necessitates imaging. EEG is usually normal in alcohol-related seizure and any abnormality should suggest an alternative diagnosis.[42]

Vitamin B1 deficiency in alcohol-related seizure is common and may be aggravated by glucose infusion and, therefore, thiamine 200 mg IV should be administered to patients with the alcohol withdrawal syndrome before injecting glucose. Mild to moderate alcohol withdrawal can be managed by supportive care.[43] A history of seizures during previous alcohol withdrawal increases the risk of a subsequent attack and such patients benefit from prophylactic AED. In mild to moderate alcohol withdrawal, seizure prophylaxis is not indicated. Patients with severe alcohol withdrawal, regardless of seizure occurrence, should receive AED prophylaxis. For alcohol withdrawal seizures, treatment with benzodiazepines (BDZ) results in reduction of seizures. Rapidly acting BDZ (diazepam and lorazepam) have higher overuse potential than slower-acting BDZ (chlordiazepoxide and oxazepam). Benzodiazepine should be chosen for primary prevention of seizure in patients with alcohol withdrawal as well as for alcohol withdrawal seizures; lorazepam and diazepam are preferred.[42] Status epilepticus may be a presentation of alcohol withdrawal syndrome and is associated with a higher risk of subsequent recurrence.[44] Lorazepam is better than diazepam, phenobarbitone, and PHT.[45] Seizure risk is proportional to alcohol intake and patients with epilepsy should be advised to abstain from alcohol.[46]

Anticonvulsants for seizure prophylaxis are usually not indicated in alcoholics; abstainers do not need them and drinkers do not take them. PHT, CBZ, and SVA, moreover, are probably ineffective in preventing withdrawal seizures. For those whose seizures are not temporally associated with drinking, those who have an additional lesion that by itself could account for seizures, or those who have epileptiform EEG abnormalities, prophylactic anticonvulsants may be required even though patient compliance is unlikely.

### Recommendation

CT scan is indicated for a first alcohol-related seizure. Change in type and frequency of seizure after 48 h of drinking calls for a CT scan. For prevention of alcohol withdrawal seizure and management of seizure in alcohol withdrawal, thiamine 200 mg IV is recommended, as well as lorazepam or diazepam.

## Renal Failure

### Evidence statement

Seizure occurs in acute renal failure (uremic encephalopathy) in 35% of patients and can be generalized tonic–clonic, myoclonic, or focal. An underlying cause should be looked for in patients with a focal seizure. In chronic renal failure, seizure occurs less commonly (<10%). The seizures in uremic encephalopathy may be due to uremia *per se*, electrolyte imbalance, or hypertensive encephalopathy. If no remediable cause is found, PHT, SVA, or phenobarbitone can be started. Status epilepticus is treated as usual. PHT is metabolized in the liver and 5% is excreted unchanged in urine. In renal failure, the therapeutic level decreases from 10–20 μg/ml to 5–10 μg/ml because of associated hypoalbuminemia, which results in increased active unbound phenytoin. Therefore, a lower dose of PHT is able to maintain the therapeutic effect.[47] CBZ, lamotrigine, and SVA are not influenced by renal dysfunction. However, in patients on hemodialysis, gabapentin, levetiracetam, vigabatrin, and topiramate may need dose supplementation after dialysis. Phenobarbitone and PHT need to be given in more than one dose per day and, besides, drug level monitoring needs to be done.

### Recommendation

In renal failure, seizures provoked by metabolic disturbances or drugs require correction of the triggering factors. Lamotrigine, PHT, SVA, and phenobarbitone are safer in renal failure than gabapentine, vigabatrin, topiramate, and levetiracetam, all of which require replenishment of dose after dialysis (class IV).

## Liver Disease

### Evidence statement

Seizures in liver disease are less common than in renal failure. Chronic liver disease rarely causes seizures unless associated with trauma, alcohol, or intracerebral hemorrhage. Seizures are reported in liver failure seen with Reye syndrome and Wilson disease. The first-line AEDs are enzyme inducers and should therefore be avoided in patients with liver failure. The therapeutic and toxic levels of PHT and SVA occur at lower serum levels in liver failure; hence these drugs require frequent serum drug level monitoring. Valproate may be idiosyncratically hepatotoxic and may cause Reye syndrome, mainly in patients less than 2 years old and suffering from metabolic diseases or mental retardation or in those who are taking multiple AEDs. Phenobarbitone and BDZ may precipitate liver failure and should therefore be avoided in liver disease. AEDs with little protein-binding ability and little liver metabolism (i.e., gabapentin, topiramate, vigabatrin, and levetiracetam) are most suitable in liver dysfunction, but there are insufficient data to assess their efficacy as monotherapy.[48] Intravenous PHT or BDZ are often ineffective, but reducing ammonia levels with the use of lactulose can stop the seizures.[49,50] Liver graft recipients experience seizures as a result of immunosuppressant toxicity (OKT-3, tacrolimus) or cellular rejection. One report on a grafted patient describes the successful use of levetiracetam as monotherapy after failure of PHT.[51] Wilson disease rarely produces seizures; d-penicillamine treatment however induces pyridoxine deficiency, which causes seizures. Treatment with pyridoxine or other copper chelators is recommended.[52]

### Recommendation

Hepatic dysfunction reduces enzymatic metabolism of AEDs and causes hypoalbuminemia. Gabapentin, topiramate, and levetiracetam are preferred in these conditions, whereas SVA and felbamate are potentially hepatotoxic and should be avoided (class IV).

## Porphyria

### Evidence statement

Seizures occur in about 10% of patients with porphyria. Phenobarbitone, PHT, SVA, CBZ, BDZ, topiramate, and tiagabine can precipitate an attack of acute porphyria by increasing the activity of delta-aminolevulinic acid. Gabapentine, bromide, magnesium sulfate, and oxcarbazepine are relatively safe in porphyria. In patients with status epilepticus, intravenous glucose, hematin, and paraldehyde should be given and BDZ may be used with caution.

### Recommendation

Seizure in porphyria may benefit from gabapentin, oxcarbazepine, or levetiracetam (class IV).

## Central Nervous System Tuberculosis

### Evidence statement

In tuberculous meningitis, seizure can occur because of encephalopathy, tuberculoma, or infarction. Seizure can be the presenting feature in 10–20% patients with CNS tuberculosis. The seizure in CNS tuberculosis may be generalized in 58%, focal in 38%, and tonic in 4% patients. Seizures are commoner in children than in adults (74% *vs* 14%). Half the patients presenting with acute symptomatic seizures may have seizures as sequelae. Long-term AEDs are not recommended in tuberculous meningitis. In a study on 136 patients with tuberculous meningitis, 63 had abnormal CT scan and seizures, 38 had seizures and normal CT scan, and 36 had no seizures and normal CT scan on follow-up; in these patients seizure recurred in 10, 7, and 10 patients, respectively, which was not statistically significant. In view of drug toxicity long-term AEDs were not recommended.[53] In the absence of any guidelines, patients with provoked seizure may be given a short course of AED for 3–6 months, after which the dose can be tapered if there is no recurrence.

### Recommendation

Prophylactic AED is not recommended in CNS tuberculosis (class III). Long-term AED may not be required in the acute symptomatic stage (class III).

## Neurocysticercosis

### Evidence statement

Neurocysticercosis (NCC) is the commonest cause of symptomatic seizure. The seizure in NCC is generalized tonic-clonic in 60%, focal in 39%, and complex partial in 29% of patients. A calcified NCC is found in 15–20% patients with seizure in endemic areas and the seizures are generally attributed to them. In these patients, seizure should be treated as is any other seizure. A few cysts resulting in seizure may respond well to conventional AED. Vesicular and colloidal cysts have been reported to respond favorably to albendazole treatment (given for 3 days to 8 weeks).[54]

A single enhancing granuloma is treated with 6 months of AED. If the seizures are controlled and a follow-up CT scan shows disappearance of the granuloma, the AED is withdrawn over 8–12 weeks. If NCC persists, an additional 3 months of AED is given, with or without albendazole, and treatment should be followed by a CT scan; if the lesion enlarges (>2 cm), biopsy is recommended.[55] Meta-analysis of cysticidal therapy in NCC has shown that treatment not only promotes resolution of the cyst but also provides seizure control.[54] In the light of these evidences, cysticidal therapy may be prescribed to these cases provided the infestation is not heavy (<100). However, there is consensus that cysticidal therapy is not indicated in calcified granuloma. In a pilot, randomized, open-label study on patients with a single enhancing cysticercal granuloma, clobazam was found to be better tolerated, safer, and more efficacious than PHT.[56] On RCT showed that 6 months of AED was as efficacious as 24 months treatment.[57]

### Recommendation

Acute symptomatic seizure in NCC requires AED at least for 6 months (class II). The decision to continue AED is based on persistence of the lesion on follow-up CT.
